# Usability Study of an Electronic Medical Record From the Nurse Practitioners’ Practice: A Qualitative Study Using the Think-Aloud Technique

**DOI:** 10.7759/cureus.41603

**Published:** 2023-07-09

**Authors:** Afnan Ali Alshehri, Abdullah Alanazi

**Affiliations:** 1 College of Public Health and Health Informatics, King Saud Bin Abdulaziz University for Health Sciences, Riyadh, SAU; 2 Research, King Abdullah International Medical Research Center, Riyadh, SAU

**Keywords:** nursing documentation, electronic medical record, nurse as autonomous practitioners, emr, usability study

## Abstract

Introduction: Ensuring the usability of electronic medical records (EMRs) is crucial for healthcare providers to offer efficient, effective, and safe patient care. Nurse practitioners (NPs) are integral to the healthcare system and are essential in managing patient workflows. However, few studies assess NPs' perspectives on how EMR usage affects workflow and patient care quality.

Method: In this study, the "think-aloud technique" was utilized for usability testing. It involves observing users (NPs) as they complete their everyday tasks on the EMR while vocalizing their thoughts and emotions. This method has been proven reliable and valid through various research, such as a systematic review.

Results: The EMR system used by NPs can create a heavy cognitive workload, have limited functionality, can lead to unintended errors, and may experience technical difficulties.

Conclusion: The EMR system used by NPs is challenging due to three main issues: high cognitive workload, limited system functionality, and technical problems. To improve the system, it is recommended to reduce the cognitive burden by customizing the user interface to fit the NPs' needs, enhancing the system's functionality by adding essential features and fixing any technical issues.

## Introduction

The healthcare industry widely uses information and communication technology due to its numerous advantages. These advantages include enhanced patient care, greater satisfaction and engagement, faster exchange of information, better diagnosis and management, and reduced costs [1]. Saudi Arabia is committed to using technology to improve healthcare, as included in its 2030 vision for the healthcare sector. electronic medical records (EMR) is a popular form of digital technology that can provide these benefits. Healthcare providers need to use EMRs effectively to ensure efficient and effective use of technology in healthcare. Advanced nurse practitioners (ANPs) are essential to healthcare provision and significantly contribute to patient management and workflow. ANPs are proficient nurses in clinical practice, education, research, and leadership [2]. It is crucial to understand how ANPs use EMR and its impact on their workflow and patient care. Analyzing challenges and solutions related to using EMR effectively in clinical practice is necessary.

According to the Organization for Standardization (ISO), usability refers to how well a product helps specific users achieve their goals in a particular context with effectiveness, efficiency, and satisfaction [3]. Jakob Nielsen defines usability as a quality attribute that evaluates how user interfaces are easy to use [4]. An application's ease of use, or usability, can be done during the design phase or after the application fully develops. The usability of an EMR system may be tested, allowing researchers to learn important information about user satisfaction and pinpoint improvement. A particularly effective technique for user usability testing is the "think-aloud" method, which means verbalizing thoughts while completing a task or handling an event. This entails watching and paying attention to users using the system and speaking aloud about their feelings and thoughts. This technique helps identify issues, difficulties with the user interface, and positive features. The think-aloud approach is a reliable and valid tool for collecting qualitative data, as confirmed by numerous researchers, including a systematic review conducted by Paz and Pow-Sang in 2016 [5].

ANPs have a legal obligation to examine, diagnose, and treat patients, which includes prescribing medication and ordering diagnostic tests. The EMR is crucial for high-quality, evidence-based patient care [6]. However, ANPs may need to become more familiar with the task-oriented usability of the EMR for their documentation and order entry, as their input was not considered during the initial phase of EMR implementation. Despite the importance of this topic, there needs to be more literature that addresses it. Therefore, this paper sheds more light on the usability of EMR through the think-aloud technique. The research question focuses on assessing the EMR's usability from the perspective of ANPs. Through this study, we hope to recommend necessary improvements and generate evidence-based data and information to enrich the literature on this subject. Ultimately, we aim to enable healthcare providers to benefit from digital technology in healthcare fully.

## Materials and methods

This research was conducted in a well-equipped office within the hospital premises. Three subjects were first used to pilot the study, and their feedback was noted for any necessary adjustments. The study participants consisted of five currently employed NPs from various departments with varying experience using the system. The study design involved a qualitative think-aloud observational approach, where participants were given seven tasks to perform on the EMR in an NP-like manner (Appendix A) while verbalizing their thought processes.

During the study, the mediator recorded both the screen and voice while observing and taking notes. Based on Nielsen's recommendation, a sample size of five is adequate for a qualitative usability study. This is known as "the 5-use rule" and is sufficient to identify nearly all usability issues in a system [4]. The aim is to reach data saturation, which occurs when all available data has been discovered and no new information can be found. Therefore, this study will involve five NPs (non-professional users) as participants, chosen through purposive sampling to include potential users who are all NPs.

Participants in the think-aloud study used a simulator medical record number (MRN) for research and education purposes. Their tasks were recorded on video during the study without showing their faces. The recorded speech was converted to text and coded accordingly. Common themes will be identified and presented with subthemes in cross-tabulations. Bar graphs will be created to display the usability results. Finally, the results are discussed in a written narrative.

## Results

During the study, NPs were asked to perform tasks on the EMR they typically do in their daily practice. They were also encouraged to share their thoughts out loud during the process. The author recorded their comments and thoughts in Table 1. These statements were then analyzed and categorized into issues or problems. The issues were further classified into sub-themes of usability issues and then grouped into common general themes. These general themes corresponded with the most common usability issues in a systematic usability study published by the Agency for Healthcare and Research Quality (AHRQ) [7]. Appendix A displays six columns from left to right. They show the task performed by the participants, participant numbers (N#), comments provided by the participants, usability issues found (listed in bullet points), the numbered sub-theme of the usability issue, and the lettered general theme of the usability problem. In Appendix A, themes are highlighted in orange using letters A to D. Each theme is then divided into sub-themes, represented by numbers. To quickly identify a participant's statement, it is coded using a letter and number corresponding to its theme and sub-theme, for example, A.1, A.2, A.3, A.4, B.1, B.2, B.3, B.4, C.1, C.2, and D. Appendix A and further Tables 1, 2, 3 contain a comprehensive breakdown of the themes and sub-themes with detailed descriptions.

**Table 1:** Frequency of Cognitive Workload Usability Issue NP: Nurse Practitioner

Cognitive Workload	NP No.	Frequency
Poor Information organization	NP1	3
	NP2	3
	NP3	5
	NP3	17
	NP5	8
Interface with the workflow	NP1	2
	NP2	1
	NP3	2
	NP3	11
	NP5	0
Click and writing fatigue	NP1	3
	NP2	0
	NP3	3
	NP3	4
	NP5	0
Unproper nomenclature	NP1	0
	NP2	0
	NP3	1
	NP3	3
	NP5	0

**Table 2:** Frequency of Poor System Functions Usability Issue NP: Nurse Practitioner

Poor System Functions	NP No.	Frequency
Poor documentation function and formatting	NP1	0
	NP2	0
	NP3	1
	NP3	13
	NP5	0
Insufficient / Irrelevant options for structured data	NP1	0
	NP2	0
	NP3	0
	NP3	6
	NP5	1
Higher chance to make errors	NP1	0
	NP2	1
	NP3	0
	NP3	3
	NP5	6
Unintegrated system with other departmental systems	NP1	0
	NP2	0
	NP3	0
	NP3	0
	NP5	1

**Table 3:** Frequency of Technical Issue NP: Nurse Practitioner

Technical Issue	NP No.	Frequency
Lagging	NP1	1
	NP2	0
	NP3	2
	NP3	1
	NP5	1
Limited access authorization	NP1	2
	NP2	1
	NP3	1
	NP3	0
	NP5	0

The participants' responses are divided into four themes. The cognitive workload refers to the effort needed to organize and design information that meets the user's needs. This includes avoiding situations where the same type of information is saved in different locations, irrelevant information is included in a tab, and scattered information makes it hard for users to find what they need. Also, the presentation of data could be more explicit and more coherent, and information is not grouped in a preferred cognitive pattern, such as lab investigation results and notes. In addition, the cognitive workload refers to the challenges that arise when tasks are not designed to fit the needs and practices of NP, which can cause compatibility issues and hinder workflow. This often leads to the need to backtrack to complete tasks. Furthermore, alerts for recommended actions lack cues or guidance, making it challenging to complete them efficiently. The following requirements are physical clicking, writing, and optional alerts. These additional tasks have increased the cognitive workload for NPs. The lack of standard and widely accepted names and terms also negatively adds to the cognitive workload.

The next set of requirements focuses on enhancing the functionality of the system. One of the major concerns is the absence of standardized documentation formats and functions for NPs across different units and specialties. It is crucial to have automated documentation functions, like autofill and dot-text, to expedite the documentation process and ensure better quality. NPs rely heavily on copying and pasting information from external sources without these functions. The current system has suboptimal data quality because of its limited options for structured data and its inadequate space for text descriptions. This can cause incomplete or inaccurate information. Moreover, there are concerns about the system's alerting functionality since it cannot generate sufficient alerts for errors, increasing the possibility of fraud and mistakes. Additionally, any user can modify notes others write, resulting in incorrect information. The description of test orders poses a problem since it does not differentiate between X-ray and ultrasound. Lastly, due to the limited duration options, there is a risk of unintentionally missing prescribed medications. Yet, the EMR system is not integrated with other hospital databases, such as the pharmacy system. This may lead to ordering medication that is out of stock. Moreover, it may not include other team members in co-signing or authorizing orders and notes.

The third issue with the system is that it frequently experiences lag, particularly during busy periods. Another concern is that no explicit standardized access control or privilege management exists. This means that different nurse practitioners may have different levels of access authorization and privileges within the system, with some being unable to request appointments or view specific investigation results within their specialty. Furthermore, the home screen may have varying layouts and interfaces.

The last theme pertains to inconsistencies in patient records, particularly in the documentation. Some notes in patient encounters may contradict the overall status of the patient. This could suggest inadequate data quality management measures or a lack of effort from users to ensure proper and complete documentation.

According to the analysis presented in Table 1, the most common usability issue in the EMR is a cognitive burden, followed by poor functionality, as shown in Table 2. Additionally, almost all participants reported experiencing technical issues such as slow response times and limited access to certain features in the EMR, as depicted in Table 3. Participants also agreed on the importance of having a list of commonly used orders and preferred qualities while noting that entering demographic data was the most straightforward task.

## Discussion

The information in the EMR needs to be clear and understandable to make informed health decisions. A user-friendly EMR should make accessing and processing information easy. If the system is not simple, it may cause problems for NPs and lead to errors [8]. This burden is evident in the literature as a failure to organize lab tests meaningfully, a failure to group related information according to preferred cognitive patterns, a lack of consecutive steps that flow, causing click fatigue, and a lack of clear naming conventions [7]. Nurse practitioners often find it challenging to maintain concentration and stay organized when working under tight timelines or in an environment full of distractions. Due to the nature of their work, NPs need a well-structured and comprehensive system that allows them to access all the information they need quickly and efficiently to effectively manage their patients' status. This system should give them easy access to all the information required to make informed decisions about their patients' health and well-being. Nevertheless, poorly presented and arranged information can be unintentionally overlooked or missed, eventually affecting patients' care [9].

The clinical notes and laboratory results are scattered throughout the system, making it difficult for participants to access them. For instance, when reviewing physicians' notes, participants must search in two separate interfaces called Active Clinical Note and EPR. Additionally, notes written in one interface may not appear in the other. Some participants also reported that the NP's notes fall under two categories in Active-Clinical-Note: The Physician or Nursing specialty clinical notes. Further, it is crucial to keep clinical notes organized as they play a significant role in continuous patient care, according to one study [6]. Misplaced or hidden notes, including investigation results and reports, can lead to errors. While laboratory results are usually structured data on the Active-Clinical-Note and/or EPR, tests performed outside laboratories are uploaded as a document format under EPR. Unfortunately, NPs are not always familiar with the information's location, and the system fails to alert users if the result document is out or still in progress. As a result, NPs spend much time and effort navigating the system to find and match unorganized information, which may affect patient care.

Studies show that ensuring tasks are compatible with a clinician's workflow reduces frustration and cognitive load [7-9]. Users expect each tab to guide them to the next necessary step and prompt them to complete tasks quickly without confusion. A user-friendly system should facilitate task completion without requiring the user to think about how or where to do it [8]. However, this study found no smooth transitions between steps, leading to a lack of user-friendliness [10]. Participants had to go back and forth to complete tasks constantly, and tabs were not visible on the screen, requiring users to scroll up and down to find them. A systematic review by Khajowei and Jaspers found that scrolling lists can interfere with a healthcare provider's workflow [11]. To improve usability, information should be presented in the same visual field [9].

Completing a task with redundant steps can be tiresome and decrease the system's efficiency and usability [8]. ANPs require a system that enables them to complete tasks with minimal clicks without compromising the safety of their practice. However, EMR necessitates additional, repetitive steps that cause fatigue and frustration among users. These steps are deemed unnecessary and excessive by participants who describe them as having too many clicks.

Improperly labeling systems or using unfamiliar words can make it harder for users to understand information and can increase their mental effort [7]. Even if the information is displayed on the screen, if it is not described using familiar and appropriate terms, healthcare professionals (HCPs) may ignore it, leading to more time spent searching for the needed information. This can also add to their cognitive workload as they try to understand what is being communicated.

The EMR system has a feature that is not usable because it does not follow the correct documentation format required by NPs. Each specialty or setting of NPs has a specific document format that they usually follow. For example, outpatient notes often use a template called SOAP, which stands for Subjective and Objective signs and symptoms, Assessment, and Plan. Notes in the emergency department are brief and have different layouts. The system should provide these options for NPs to standardize documentation and cover all aspects of patient examination and plan. Unfortunately, due to the poor design of the documentation function, NPs must use external sources like Word files to create their preferred documentation format and then copy it into the free-text bar under notes. However, imposing strict regulations and relying too heavily on structured data can negatively affect data quality and cause the HCP system to resist adoption [12]. This resistance can be further exacerbated by limiting the HCP's abilities [7]. The system does not provide relevant or adequate structured data. Forcing the HCP to choose from a given option may not adequately describe patients' conditions, resulting in poor data quality documentation [13]. Therefore, the NPs will ignore these types of documentation and, instead, will copy and paste into the free text.

The current system is ineffective at minimizing errors or protecting patients from harm. Nurse practitioners have reported encountering incorrect or unavailable orders when making choices. For instance, some radiology types are not specified, leading to errors. Healthcare providers aim to communicate patient data efficiently within their organization. Integrating EMR with other systems, such as pharmacy databases and laboratories, can improve communication and enhance patient care quality. However, a recent study found that the integration of these systems is poor [14]. For example, some medication order lists were not updated with the current stock availability, as reported by one participant.

The NP has encountered a problem that disrupts their work and affects patient care. Whenever the NP orders a medication the pharmacy does not have, the pharmacist rejects it and asks the NP to make changes. Therefore, we suggest creating a personalized EMR interface for each ANP based on their specialty, needs, and cognitive pattern to solve this issue. This will enhance the usability and efficiency of their practice and improve patient care quality and safety.

While the think-aloud technique can provide valuable insights into human behavior and cognition, it can also have drawbacks. For example, self-consciousness can affect how accurately individuals express themselves and alter their behavior and thoughts. It can also make it challenging to keep them on track and potentially disrupt others in the same environment [15]. However, despite these challenges, the technique remains helpful in understanding human thought processes.

## Conclusions

Advanced nurse practitioners require an EMR system with improved usability. The current system only partially facilitates their practice, resulting in errors and patient harm. A detailed analysis has identified specific usability issues. Recommendations include a customized user interface to reduce cognitive burden, clinical documentation forms and functionality to support NPs' practice, and necessary technical operations and fixes.

## Appendices

**Table 4:** Appendix: Coding table

Coding table					
Task	n	Participants thoughts	Usability Issue	Sub-theme	Theme
Review pt. demographics	n1	"I have no comments here" D	No problem noticed	1. Poor information organization and display 2. Poor design and interference with workflow 1. Lagging	A. Cognitive workload C. Technical D. contrary to issues found
	n2				
	n3	"Very clear" D “there is also an icon on the side with all of the information which I find is too much information for me” A.1	Clear Not useful overly clutter information		
	n4	“okay now none of the tabs are playing up hold on” C.1 "I tend to overlook them because I'm usually looking for tabs over here " A.2 “it would be easier if either the tabs are all inclusive of everything and these icons are removed because it's duplication” A.2	Technical lagging Duplicate & not inclusive information Poor design & information organization		
	n5	"it’s so easy" D	Easy to find		
Review patient overall condition summary	n1			1. Poor information organization and display 4. no use of common language 2. Lagging	A. Cognitive workload C. Technical
	n2	“patient overall condition, sometimes if the patient new we will see it from the referral” A.1	No clear concise place to access certain information		
	n3	“there is some headlines that for example here like cancer stage profile it may indicate that this patient is having cancer but there is no data inserted so he is not, so I find it kind of confusing” A.1	Confusing Poor design and information organization		
	n4	“not like a clear concise area” A.1 “in the tabs you have patient history but most of the time it's just a bunch of diagnosis” A.1 “not very clear in terms of medical” A.4 “as far as history that that's not necessarily a history” A.1	No clear concise place to access certain information Irrelevant information Improper nomenclature		
	n5	“So slow” C.1 “condition summary either to go to EPR or active clinical note” A.1 ”it takes too long to review patient in one visit” A.1	Technical lagging No clear concise place to access certain information Time consuming		
Review physician & NP notes	n1	“There’s other problem if the physician are using the ERP it will not be appear in the active clinical note. So we have to go back to the EPR then nursing assessment and we will find the note here” A.1, A.2	Scattered information, no concise place to find one information Design interfere with workflow	1. Poor information organization and display 2. Poor design and interference with workflow 1. Lagging	A. Cognitive workload
	n2	“most physician they will do it through EPR from here. And if they will do it from EPR it will not show in active clinical note” A.1, A.2 “if I wrote a note last week and I will update my note for example it will appear as I did it today” A.1	No specific place for same information Chance of missing information Not organized chronological order		
	n3				
	n4	“the notes are not necessarily in chronological order” A.1 “sometimes people cannot find the notes because they're not looking in the right place” A.1	Not organized chronologically No clear a concise place to find certain information		
	n5	“some of the note will appear with speciality some of the note without speciality” A.1 “some of the nurse practitioner their note in nurse section some of the nurse practitioner to the physician note” A.1 “is very difficult to review all the data for multiple visits, its take too long” A.1	No categorization of presented information No concise place for an information Time consuming to understand information presented		
Review lab results	n1			1. Poor information organization and display 1. Lagging	A. Cognitive workload C. Technical
	n2				
	n3	“now we can take a nap until..” C.1 “too many icons I don’t need, and it looks like cluster” A.1 “on the left you can read lab cumulative results then next to it cumulative lab results, so I don’t know what it the difference between them to be honest” A.1 “icon without any information… so why do I have it” A.1	Poor design and organization of information Looks clutter Confusing Unnecessary duplicating tabs		
	n4	“there's a couple of places so again under active clinical note it can be confusing to access” A.1 “there's a duplicate tab” A.1 “haem results which I find very complicated” A.1 “very confusing” A.1 “it should be in chronological order by date” A.1 “it doesn't seem like you can just pick okay CBC and then you can put it in order of how when it was done last things like this” A.1	Failure to group information in preferred cognitive pattern More than one place to access same information Confusing Duplicating tabs Not arranged well, not chronological		
	n5	“lab test result either from here laboratory or will go from EPR so this is another problem” A.1	Scattered information		
Review radiology & other documents tests	n1	“if the patient have done ECHO or Holter or stress test then we have to go here then clinical documents” A.1 “CT coronary Angio or MRI it’s going to be from here, ECG its suppose to appear from this icon.. this is one problem that I face because it’s not supported in my account” A.1, C.2 “usually if the patient has a Holter report it going to take time to open so for example here I will open his echo. With the Holter the gray screen it will take time and it will not open immediately” C.1	Information can be found in more than one place Limited authorization access Slow response	1. Poor information organization and display 1. Lagging 2. Limited authorization & access	A. Cognitive workload C. Technical
	n2				
	n3				
	n4	“Radiology for the most part it's fine” D “very confusing because if you have un-resulted okay the whole point is that you're waiting for your results so why are you going to go to un-resulted tab to view un-result” A.1	Non-useful information		
	n5	“we don’t know if the result came or not” B.3 “It will not appear hear will appear in EPR document” A.1 “some of the referred tests will come in the lab section, some of the test will appear here in document” A.1	Failure to alert for resulted tests No specific and concise place for certain information		
Enter an order (lab, meds, radio)	n1	“the good thing here we have a favorite, so we can add our favorite” D “we have to fill this boxes” A.3 “I have to write here anything any comment, save then update then update the issue here that we have too much updates A.3. Here asking for brief history an update” A.2, A.3	Preferred common orders made it easy and fast to order Too many physical clicks and writing Interference with workflow of task	1. Lack of information organization 2. Interference with workflow 3. Too many clicks and writings 4. no use of common language 1. No proper documentation format and functions such as automated documents 3. chance of error harming patient 4. no integration between systems 1. Lagging	A. Cognitive workload B. poor functionality C. Technical
	n2	“usually I can do the preference the order set the preference lab the most the things that mostly use or order” D			
	n3	“Is taking long time as you can see “ C.1 “I find it very tiring and why do I have many boxes to fill we can it’s not mandatory or important” A.3 “now put the dose 200 when needed so prn it not there on the list, so you can only see on the list phrases there is no use of common language” A.4 “also, here another box and here another box so it is tiring and there is save and close and there is update on the right so I think we all agree that this can be much easier with minimum clicks than the current situation” A.3	Technical lagging Too many unnecessary writings and clicks No use of common language Does not match workflow Tiring Time consuming		
	n4	“a lot of orders get discontinued too early and then what happens is that the patient goes a couple of days without the medication” B.3 “other issue is that you have to put order notes a couple of times” A.3 “other issue is it’s PRN it's not scheduled” B.1 “as soon as you click that is PRN it should already indicate what it is PRN for” B.1 “put order notes what you're putting it for, and then you have to put again what's the indication for, so it's duplication, which is unnecessary” A.3 “there's already some pre-selected common Labs here which is fine” D “why am I putting an order note for a CBC! I don’t think there’s needs to be a note for every single thing that you're doing that's part of being a provider and you're collecting routine Labs” A.3 “again, you have to put your password… I don't think that it needs to be for every single order that you're doing” A.3 “this is just order entry but again I can't see history of what I put in what I didn't so that I don't find to be user-friendly” A.2 “we just figured out where the orders are. You have all orders and then you have them broken down by what type of order, but even then, you're still missing a few” A.2, A.1 “it should still show up that it's pending” A.1 “consultations are definitely something that should be under its own separate tab” A.1	no notes automation make work longer and tiring Too many unnecessary writings Does not match workflow Missing info not user friendly failure to alert poor and unappropriated tabs organizations		
	n5	“some of the medication when order paracetamol like 125 specific with order, pharmacy will reject it and they will call for its unavailable” B.4 “lab, we don’t have any issue with lab for ordering its easy with all specific” D “For radiology, like for hip, ultrasound, we don’t know is it bilateral hip joint x-ray or ultra sound, like sometimes we order ultrasound but by mistake because not specify here if it x-ray, they should specify bilateral hip joint x-ray, or hip joint CDH xray” B.3	Is not integrated with other systems such as pharmacy Errors Laboratory good Radiology wrong orders are available in system		
Write your clinical note	n1			1. lack of information organization and grouping 2. Interference with workflow 3. Too many physical clicks and writings 1. No proper documentation formats Irrelevant or unavailability of provided adequate options affecting data quality 3. chance for error or harm	A. cognitive workload B. Poor system function
	n2	“if I didn’t do authorized another physician can enter my note and changed my note” B.3	Chance for error or fraud		
	n3	“ I will go to the note then physician or nurse I will expand I want to add my note there is no box for that there is no box to fill no box to add, so in order to add the note I have to go to the left of the screen entry type and choose nurse practitioner note then press new then I can add my note there is different page for that so if I don’t know the system I will be lost” A.2 “you can write the note you want but the thing is that its blank space there is no vitals no information any data about this patient I need to review or see so this is one big problem… maybe I will miss very important vitals or something” A.2, B.1 “and again, we will go through updates and save” A.3	Compatibility with workflow No proper documents function		
	n4	“I don't have a consultation note from my specific specialty” B.1 “new consultation so you click on it and then new, and over here are the parts of the note that you can use, to fill out your notes. I personally do not find this user-friendly in any way shape or form” B.1, A.2 “I just have a template that I use, and I copy and paste” B.1 “it doesn't make sense for what an HPI portion of the note is. HPI is going to be your history of present illness so it's going to be a good paragraph” B.2 “this should already be filled in, B.1 and then it should be a space where I can just click and put my info in” B.2 “this should automatically be tabulated B.1…you shouldn't have to go back in and include that information A.2 because it should be carried over into your note” “this stuff should already be in your note carried over from the demographics and from the patient history B.1. this also creates duplicate work and makes your notes take twice as long than what they should” A.2 “vitals also should be pulled in, you shouldn't have to copy and paste” A.2, B.1 “the physical exam it's not completely inclusive let's say I'm listening to their lungs I want to put clear to auscultation bilaterally I can't, there's not a way for me to input that” B.2 “And then when you update it in your note you see this part in the in the physical exam of what you put, but again it's not following it's just like a piece of it in your note” B.1, A.2 “I want to add something else to you know assessment plan, blah blah blah whatever whatever, okay you save it and you update it, now it's showing up as a plan! but why?!” B.1, B.2 “so the layout the template of the note just needs to be one template where you can just go to the tab to the different spots and input the data instead of clicking and piecemealing the note, because then it's not clear and it's also not easy to use for the provider” B.1, A.2 “the easiest way to do this again is I click on progress note and I copy and paste my template that I use which is a soap note most people use soap note, that is the template that should be included here subjective objective assessment and plan it doesn't have that” B.1 “There's an order tab in the progress note not really sure why! then consent for anaesthesia and sedation! Okay so we're using that as a progress note?! How come?! they’re included as a sub section for progress note, instead of their own separate document” B.2, A.1	Time consuming Copy and paste errors Depends on memory cognitive workload Does not match workflow not providing necessary templates Not relevant Poor organization and layout		
	n5	“should be once the patient in the clinic it should be unified, and its only outpatient note not many option” B.3 “it’s free text it’s better to be unified with task bar” B.2 “we should save the note as authorized, so no one will enter my note and correct the note, the problem here that affect the patient documentation that anyone can enter my note and add in my progress note without informing me… the first user they cannot know who enter his note or correct his note” B.3 “there is no box for co-sign the note… it should be send direct to the consultant or the MRP to sign the note review the note of trainee… the consultant he doesn’t know the note need to be co-sign” B.4	Allowing too many options without standardization Room for intentional or unintentional mistakes No co-signs options because accounts are cannot be liked	2. No adequate format for documentation 3. Chance for error or fraud 4. No integrated system	
request a follow up or make an appointment	n1	“my user doesn’t support giving appointment, so I have to ask the physician to book for him” C.2		1. poor information organization and design 2. no compatibility with workflow 4. No use of common language 2. Irrelevant / inadequate structured data provided 3. Chance for error 2. Limited access & authorization	A. cognitive workload B. poor system function C. Technical D. contrary opinion
	n2	“I can’t do follow up appointment if I want unless if I can do direct booking in the clinic if there is a slot” C.2			
	n3	“appointment for me for some reason with my account I can’t do that I don’t know why” C.2			
	n4	“I have not tried to request an appointment or do an appointment so I'm going to just see … I'm assuming that… but since I can't even pull that up.. lets see..” A.1 “I’m not really sure what B.O is! no idea” A.4 “resource I guess can be confusing but once you actually click on it you realize it's the provider so maybe this should say provider instead of resource” A.4 “that seems pretty straightforward” “although I don't know why you have retinoblastoma for a session type!! or uveitis!! for session type, that would probably be more for the comments of why you're Consulting them or why they're doing an appointment” B.2 “not really sure what it's looking for I'm just seeing cuz I have no idea” A.1 “it tells me that it's been added that the referral has been added yes, it's been added but where? you don't see the time! you don't see where! you don't see what’s the schedule for the person! so this is not user-friendly… so I’m not really sure about that” A.2	Not proper use of terms confusing Not relevant A lot of wandering throughout Time consuming to figured out Not clear Not easy to complete task Does not assure hcp its done correctly Mistakes Not match workflow		
	n5	“appointment its easy” D “another problem that I can choose follow up or OPD referral the new referral, it should be once the patient already seen by paediatrician before it should be only appear the follow up, if the patient new to the clinic it should appear only referral, so some of the physician will abuse the system and put most of the patient as new referral and this is will make the data of the consultant is wrong” B.3 “allowing more option for the appointment is not good” B.3	Easy Too many options confusing Chance to abuse system or fraud		
